# Trauma-informed Interventions in Early Childhood Education and Care Settings: A Scoping Review

**DOI:** 10.1177/15248380231162967

**Published:** 2023-04-12

**Authors:** Yihan Sun, Claire Blewitt, Victoria Minson, Rachael Bajayo, Lee Cameron, Helen Skouteris

**Affiliations:** 1 Monash University, Melbourne, VIC, Australia; 2 Australian Catholic University, Melbourne, VIC, Australia; 3 Alannah & Madeline Foundation, Melbourne, VIC, Australia

**Keywords:** trauma-informed, early childhood, childhood trauma, multi-tiered, training

## Abstract

Trauma in early childhood is a significant public health concern. Early Childhood Education and Care (ECEC) services are uniquely positioned to buffer the negative impact of early childhood trauma on children. This scoping review synthesized studies evaluating trauma-informed interventions in ECEC settings through a systematic search of four relevant online databases (PsycINFO, Medline, ERIC, A+ Education). Fourteen studies met the inclusion criteria, with 12 ECEC center-based trauma-informed interventions evaluated. Types and components of trauma-informed interventions, outcomes, and measures are presented. Findings suggest that trauma-informed interventions in ECEC settings are nascent but growing. Increasingly, programs are adopting multi-tiered system of support to address early childhood trauma, with these models suggesting promising results. The predominant focus of ECEC center-based trauma-informed interventions was upskilling teachers through training and coaching, with studies focused on assessment of teacher-level outcomes. Child, organization, and caregiver-level outcomes are not explored to the same extent, with evaluation of organizational outcomes relying predominately on qualitative methods. Whilst the short-term outcomes of trauma-informed approaches in ECEC have been examined, longer-term impacts and the causal mechanistic pathways of such programs have yet to be explored.

## Introduction

### Trauma in Early Childhood

Early childhood is a critical developmental period that shapes children’s long-term social, emotional, and physical health (Shonkoff & Phillips, 2000). Trauma at this sensitive period of child development is a significant public health concern that can have multiple detrimental impacts on children’s development and health. Though no universally agreed terminology exists (Hanson et al., 2018), trauma, in this review, refers to an emotional response to a terrible event that leads to physical or psychological harm for an individual (American Psychological Association [APA], 2022). Childhood trauma encompasses childhood maltreatment by adults (e.g., neglect, abuse), traumatic experiences formally identified in DSM-5 criteria (APA, 2013), and adverse childhood experiences [ACE] (Feliti et al., 1998). These traumatic experiences can be either acute or chronic, directly experienced or witnessed, that overwhelm a child’s ability to cope (Iachini et al., 2016). Extensive research evidence points to the deleterious consequences of childhood trauma for children, families, and society (e.g., Dye, 2018; Kempke et al., 2013). A secondary analysis of data from the Fragile Families and Child Wellbeing Study in United States found that 55% of preschool children had experienced one ACE and 12% had experienced more than three (Jimenez et al., 2016), while another indicated that 26% of children witness or experience a traumatic event before they turn four (Briggs-Gowan et al, 2010). The COVID-19 pandemic has resulted in increasing risk for childhood trauma (e.g., Cappa & Jijon, 2021).

Experiencing trauma without a supportive trauma-informed relationship and system in place may generate toxic stress, leading to intergenerational transmission of disparities in educational achievement and health outcomes, with potential consequence for the pathogenesis of adult disease (Shonkoff et al., 2012). Childhood trauma can harm children’s neurodevelopment and functioning (e.g., brain, nervous system, endocrine system) (De Bellis & Zisk, 2014), social and emotional competence (Powers et al., 2015), behavior (Copeland et al., 2007), and executive functioning (De Prince et al., 2009). To buffer the potential negative effects of childhood trauma, a joint effort of services and support around the child (i.e., family, Early Childhood Education and Care (ECEC) centers, health providers) is needed. An increasing number of programs have been developed to respond to trauma-impacted young children’s needs. However, an interdisciplinary review of trauma-informed practices over the past two decades suggests that researchers and practitioners are just beginning to engage with trauma-informed practices in educational settings (Thomas et al., 2019).

### Trauma-Informed Approaches in ECEC

ECEC centers include long day care and childcare centers, kindergarten, nursery, preschool, and early learning centers where young children are cared for and educated by early childhood teachers (Organization for Economic Cooperation and Development [OECD], 2021). The term “teachers” is used throughout this article and refers to early childhood practitioners, with a breadth of qualifications and skillsets, who work with children in the ECEC environment. The Bioecological Model views child development as a complex system of relationships affected by multiple levels of the surrounding environment, from immediate settings of family and school to broad cultural values, laws, and customs (Bronfenbrenner & Ceci, 1994). Within this model, ECEC settings are situated within the microsystem, one of the most immediate environmental settings that play a vital role in child development. Factors such as the physical environment, teacher–child interactions, teacher beliefs and practices, policy and philosophy of centers are thus vital in responding to children who have been exposed to traumatic experiences.

Trauma-informed care, programs, systems, approaches, and environments are developed to mitigate the impact of trauma. An intervention is trauma-informed if it demonstrates a *realisation* of the widespread impact of trauma and understands potential pathways toward recovery; a *recognition* of the signs and symptoms of trauma in individuals and groups; a *response* that involves full integration of knowledge about trauma into policies, procedures, and practises; and efforts to prevent *re-traumatisation* of individuals and groups (Substance Abuse and Mental Health Services Administration [SAMHSA], 2014). Several multi-tiered frameworks have been developed to promote trauma-informed care (e.g., multi-tiered system of supports, SAMHSA’s (2014) Multi-tiered Model). In this review, we define Tier 1 as programs that upskill the ECEC workforce to benefit all children regardless of trauma experience (e.g., training and capacity building). Tier 2 includes targeted programs developed for children at risk of trauma or showing signs of related socio-emotional issues without formal screening (e.g., therapist-led play session delivered in the classroom for children who may have witnessed or experienced trauma). Tier 3 refers to individualized, intensive support for children with significant concerns (e.g., consultation for children with posttraumatic stress disorder by a psychologist; clinical therapy). A program is multi-tiered if more than one level of support is provided (SAMHSA, 2014).

### Rationale for the Current Study

To our knowledge, previous reviews have not synthesized the evidence relating to trauma-informed interventions in ECEC settings in a comprehensive and systematic way. Recognizing the unique position of ECEC centers in responding to trauma-impacted young children and the paucity of such synthesis, a review focused on trauma-informed intervention in ECEC appears timely and warranted.

The aims of this systematic scoping review were to: (a) identify, describe, and examine the type of trauma-informed interventions that have been evaluated in ECEC settings; (b) examine child, teacher, classroom, and/or organizational outcomes that are associated with trauma-informed interventions in ECEC settings; (c) explore the measures that have been used to assess trauma-informed program outcomes; (d) explore the methodological limitations of research investigating the impact of trauma-informed programs applied in ECEC settings; and (e) make recommendations for policy, practices, and future research.

## Method

The aim of this systematic scoping review was to explore the extent and scope of literature on trauma-informed intervention in ECEC. In contrast to a systematic literature review that seeks to answer specific and clearly defined research questions, a scoping review is suitable to determine the scope or coverage of a body of literature on a given topic, provide clear indication of the volume of literature available, and an overview of the research focus. A scoping review enables one to examine how research has been conducted, identify, and analyze knowledge gaps and informs practice in the field and future research (Munn et al., 2018). This review was conducted in accordance with the Preferred Reporting for Systematic Reviews and Meta-Analysis (PRISMA-ScR) guideline for scoping reviews (Tricco et al., 2018).

### Search Strategy and Study Selection

Peer-reviewed papers in English were sourced from four relevant online databases: PsycINFO, Medline, ERIC, A+ Education. Publication dates were limited from 2011 to June 2022. Following SAMHSA’s Concept of Trauma and Guidance for a Trauma-informed Approach (2014), there was increased use of trauma-informed approaches and programs within educational settings. Hence, the search window (2011–2022) is appropriate to comprehensively capture relevant, contemporary research. Three groups of key terms were combined to yield a comprehensive set of papers as shown in Supplemental Table S1. Covidence was used to manage the search results. Manual searching of references cited in selected papers and relevant reviews of school-based trauma-informed intervention programmes were undertaken, and suitable papers included.

### Inclusion and Exclusion Criteria

Trauma-informed approaches have been increasingly embedded within ECEC programs. The search was limited to studies conducted in center-based ECEC settings, including kindergartens, preschools, and childcare services, for children from birth to age six. We recognize that center-based ECEC differs from home-based ECEC programs, such as family daycare, in several respects, including the environment and professional development available, which may influence trauma-informed intervention development, implementation, and evaluation. While it is important to understand the availability and effectiveness of trauma-informed intervention in non-center based ECEC settings, this was beyond the scope of the current review. The interventions of interest were those that explicitly targeted children who had experienced, were experiencing, or were at risk of experiencing trauma, or their early childhood teachers, or early years organizations who are preparing to be trauma-informed at a service level. Studies were included if they described a program that explicitly focused on the provision of trauma-informed care or practices. Programs may target the teacher, child, classroom and/or organization, and at least one child, teacher, classroom, or organization-level outcome must be measured post-intervention. Study design was not restricted. For example, randomized controlled trials, quasi-experimental studies, pre-post designs, single subject designs, qualitative and mixed-methods studies were eligible for inclusion (for information please see Supplemental Table S2).

### Review Procedure and Data Extraction

**Figure 1.**
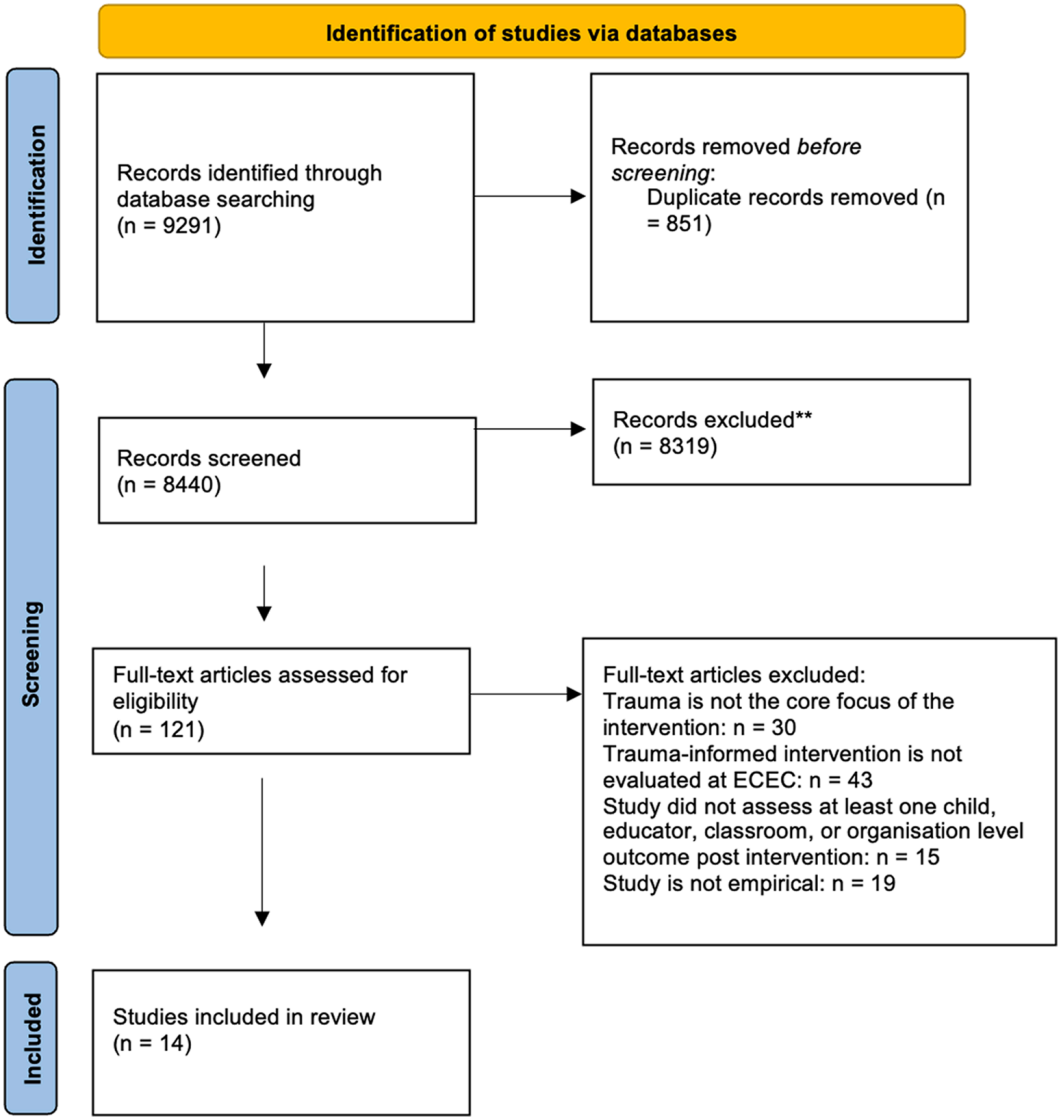
Preferred reporting items for systematic reviews and meta-analyses chart.

As shown in Figure 1, after removing the duplicates, 8,440 publications were evaluated at the title and abstract screening stage for potential inclusion against the criteria by two researchers [*YS & CB*]. Any discrepancies in co-screening were resolved through discussion. A total of 121 papers were read-in-full to further assess the eligibility for inclusion, with 14 articles included in the review. The excluded studies focused predominantly on medical or dental trauma, and trauma-informed interventions in school settings. The following data were extracted from each study to allow comparison: (a) Study design (study aim, setting, theoretical framework, study design, location); (b) Characteristics of child and teacher participants (number, age, gender, ethnicity); (c) Intervention description (intervention name, format, frequency and duration, lead, caregiver involvement, fidelity); (d) Outcome measures, informant, and findings.

In order to classify each intervention as Tier 1, Tier 2, Tier 3, or multi-tiered, the following components of the intervention were extracted, informed by the SAMHSA’s (2014) six principles and Avery et al.’s (2021) review of school-wide trauma-informed approaches: inclusion of staff PD (professional development/training), organization-level change (e.g., center-level capacity-building), cultural considerations/adaptation, family involvement, inclusion of therapy, coaching, and explicit program delivery.

## Results

### General Characteristics of Included Studies

Fourteen studies were included in the scoping review. The characteristics of each study are reported in Table 1. All were published between 2015 and 2022 inclusive and conducted in the United States. Seven studies reported quantitative data only [reference number in Table 1: 2, 3, 5, 7, 11, 13, 14], with one study using a randomized controlled trial [3], two studies adopting a pre-test post-test control group design [5, 7] and four studies using single-group pre-test post-test design [2, 11, 13, 14]. One study relied on qualitative methodology [1], and six adopted a mixed-method design [4, 6, 8, 9, 10, 12]. Among these, two studies adopted a single group pre-test post-test design [9, 12], and one study each reported using a cluster randomized clinical trial [8], randomized controlled trial [10], controlled, pre-test post-test design [4], and feasibility evaluation using a mixed-methods approach [6].

**Table 1.** Study Characteristics.

No.	First author (Country)	Design	Conceptual Framework	Setting	Participants	Intervention	Duration	Receiver	Lead	Limitations
1	Douglass (2021) U.S.	Case study	System Theory	5 urban ECE programs	5 ECEC programs team members + BSC staff and coaches	The Breakthrough Series Collaborative (TIC BSC) Initiative	1–2 year period	Organization	Certified “Improvement Advisor”	– Turnover impacted some interviews and one service dropped out.
2	Orapallo (2021) U.S.	Longitudinal pre-post design	ARC Framework; Developmental Ecological Model	42 preschools and elementary schools	2418 staff	Trauma Smart	20 hr of training delivered in 10 × 2 hr sessions each month of the academic year.	Teachers, administrative personnel	Masters-level clinicians	– Measured one component of many, thus difficult to gauge whether the shifts in attitudes were a result of the training or the larger program.
3	Tucker (2021) U.S.	Pre-test post-test control group design	Attachment and Regulation Theory	3 state-funded preschool classrooms, 2 Head Start classrooms	189 preschool children	Sunshine Circles	1+ session per week, 20–30 min per session.	Children	Teachers, mental health professionals	– Rater bias (teachers/parents).
4	Gilles and Carlson (2020) U.S.	Pre-test post-test control group design	ARC Framework	2 Head Start Sites	5 teachers, 5 teacher assistants, 106 children, 1 preschool director, 3 mental health consultants, 1 building supervisor, 106 primary caregivers.	Trauma supplement intervention	4 months	—	—	– Low levels of fidelity, limited duration and intensity of intervention. – Non-randomized selection of site and non-blinded measures could introduce bias.
5	Tabone (2020) U.S.	Pre-test post-test control group design	ARC Framework	74 pre-K, K, and 1st-Grade classrooms in 11 schools	74 classrooms	Trauma-informed Elementary Schools (TIES)	3 school years	ECT	Resource liaisons	– Relied on single observer using CLASS tool – Substantial drop-out – Did not use randomized controlled design.
6	Lipscomb (2019) U.S.	Mixed method feasibility	Research on resilience	Online	17 ECT	Roots of Resilience	Training: 6 modules Online coaching: 6 sessions	ECT	Trained coaches	– Potential for bias in self-reported measures.
7	Rishel (2019) U.S.	Pre-test post-test control group design	ARC Framework	51 classrooms (including pre-K) in 11 schools	—	TIES	Two school years	ECT	Resource liaisons	– Relied on single observer – Substantial drop-out – Did not use randomized controlled design.
8	Whitaker (2019) U.S.	Cluster randomized clinical trial	NR	26 preschools	96 ECT	Enhancing Trauma Awareness (ETA) Course	Six sessions (12 weeks)	ECT	Certified trainers	– No active control group, participants were not blind to study condition, all surveys self-reported.
9	Woods-Jaeger (2018) U.S.	Single group Pre-test post-test design	High-quality caregiving with attention to social and contextual factors	Infant and toddler classrooms at Head Start	86 children predominately Black (79.1%) 20 teachers: 45% White, 45% Black 25 parents: 80% Black, 92% female	2Gen Thrive Preventive Intervention (Classroom Theraplay and DBT4P)	Classroom Theraplay: 2x week for 7 weeks, 28 sessions total	Classroom Theraplay: children DBT4P: parents	Classroom Theraplay: Licensed clinical social workers or music therapists DBT4P: Psychologists	– No control groups – Variability in timing of pre and post assessment limited statistical analysis of quantitative data.
10	Tucker (2017) U.S.	Pre-test post-test control group design	NR	6 Preschool sites	206 preschool children Age: 3–4 Diversity: 9 children qualified for special education services, 52% English language learners	Sunshine Circles	1+ sessions per week, 20–30 min per session.	Children	Teachers who received training from a certified trainer	– Non-randomized design – Potential for bias in rating tools.
11	McConnico (2016) U.S.	Single group pre-test post-test design	Supportive trauma interventions for educators (STRIVE) Framework	All kindergarten, first, and second grade classrooms at a pilot school	250 children 12 educators	STRIVE	10 hr training	ECE	NR	– Small number of teachers makes it difficult to evaluate statistically significant changes in efficacy.
12	Perry (2016) U.S.	Single group pre-test post-test design	NR	1 Title I educational institution serving children ranging from Pre-K – G8	32 teachers and/or administrators 19 families	The New Haven Trauma Coalition (NHTC)	Training: 2 days Cognitive Behavioral Intervention for Trauma in the Schools program: 10 weeks	Training: teachers and/or administrators Care coordination: Families	Care coordination: A masters level care coordinator	– Care coordination component unsustains. – The pilot school opted to refer students exhibiting moderate to high levels of negative behavior in the classroom.
13	Shamblin (2016) U.S.	Single group pre-test post-test design	Early childhood mental health consultation	Pre-K classrooms	11 teachers	The Partnerships Program	—	Preschool teachers	Consultation: Trained consultants Workforce Development: trainers	– Targeted to highly impoverished rural school, may lack large-scale generalizability – Small sample size – Lack robust control.
14	Holmes (2015) U.S.	Single group pre-test post-test design	ARC Model	Head Start	81 children Age: 31–76 months, *M* = 4.25 years Ethnicity: 39% African-American, 15% non-Latino White, 8% Latino/Latina, 3% other, data unavailable for about one-third (35%) Gender: 36% female	Head Start Trauma Smart (HSTS)	1–2 years	Training: All staff (e.g., teachers, parents, administrator, receptionist, bus drivers) Intensive individual trauma-focused intervention: Referred children Classroom consultation: Teachers and students	Training: HSTS Therapists (Master level) Trauma-focused intervention: Masters-level therapists with degrees in social work or counseling Classroom consultation: A mental health clinician	– No control groups – Fidelity not assessed – Urban setting only.

*Note*. NR = Not reported; ARC = The Attachment, Regulation and Competency; ECT = early childhood teacher; ECE = early childhood educator; TIC BSC = Trauma-Informed Care Breakthrough Series Collaborative; DBT4P = Dialectical Behavior Therapy Skills Training for Parents; CLASS = Classroom Assessment Scoring System; ECEC = Early childhood education and care; No. = study number.

Eleven studies [1–7, 9, 11, 13, 14] reported an underpinning theory or framework that informed the interventions. It is notable that five studies [2, 4, 5, 7, 14] reported the adoption of Attachment, Self Regulation, and Competency (ARC) Framework, which aims to support trauma-impacted children through strengthening the caregiving system, building capacity in self-regulation, and developing children’s resilience (Blaustein & Kinniburgh, 2018). System theory, which highlights the significance of change across organizations instead of only at the individual level, was used to inform the development of Trauma-Informed Care Breakthrough Series Collaborative (TIC BSC) [1]. Early Childhood Mental Health Consultation, which aims to strengthen teachers’ capacity to promote children’s social and emotional learning, and mental health wellbeing, informed the Partnerships Program [13]. Other studies reported interventions were informed by relevant research and theory, including attachment, research on resilience [6], and high-quality caregiving with attention to social and contextual factors [9].

### Types of Trauma-Informed Interventions in ECEC

Twelve trauma-informed interventions were captured, with three evaluated in two studies respectively (Trauma-informed elementary schools [TIES] [5, 7], Trauma Smart [2, 14], Sunshine Circles [3, 10]). Detailed components of each intervention are shown in Table 2. Five programs were classified as Tier 1 (Sunshine Circles [3, 10]; Enhancing Trauma Awareness Course [ETA] [8]; Roots of Resilience [6]; Trauma Smart [2]; Supporting Trauma Interventions for Educators [STRIVE] [11]), as they focused on building the capacity of teachers, administrators, and organizations to benefit all children, without assessing children’s experiences of trauma or providing further targeted and/or intensive support. One intervention was classified as Tier 2 (2Gen Thrive), developed in response to the high levels of early life trauma among parents and children within Head Start services (Woods-Jaeger et al., 2018). This program supported families at risk of toxic stress (i.e., high levels of ACEs), through Classroom Theraplay including therapist-led activities delivered to children in collaboration with teachers, and Dialectical Behavior Therapy Skills Training for Parents (DBT4P) consisting of psychologist-led group sessions aimed at improving parents’ stress-management, and emotional regulation (Woods-Jaeger et al., 2018). This review did not identify any studies that evaluated a stand-alone Tier 3 intervention. However, Tier 3 intervention components were embedded in four multi-tiered interventions (Table 3).

**Table 2.** Intervention Components.

	Intervention Components										Outcome Assessed			
Tier	Intervention	Staff PD	Coach	Organizational change	Cultural	Family Involved	Assess Trauma	Therapy	Explicit Program	Child	Teacher	Room	Organization	Study
Tier 1	Sunshine Circles	✓	✓						✓	✓	✓	✓		Tucker
	ETA	✓									✓	✓		Whitaker
	Roots of Resilience	✓	✓								✓			Lipscomb
	Trauma Smart	✓	✓	✓							✓			Orapallo
	STRIVE	✓	✓						✓		✓	✓		McConnico
Tier 2	2Gen Thrive		✓		✓	✓	✓	✓	✓	✓		✓		Woods-Jaeger
Multi-tiered	HSTS	✓	✓		✓	✓	✓	✓		✓		✓		Holmes
	Trauma supplement intervention	✓		✓			✓			✓	✓	✓	✓	Gilles
	Partnerships Program	✓	✓		✓		✓	✓	✓	✓	✓	✓		Shamblin
	TIC BSC Initiative	✓	✓	✓	✓	✓					✓	✓	✓	Douglass
	TIES	✓	✓	✓		✓		✓	✓			✓		Tabone and Rishel
	NHTC	✓	✓	✓		✓	✓	✓		✓	✓		✓	Perry

*Note*. STRIVE = Supportive trauma interventions for educators; ETA = Enhancing Trauma Awareness; HSTS = Head Start Trauma Smart; TIC BSC = The Breakthrough Series Collaborative; TIES = Trauma-informed elementary schools; NHTC = The New Haven Trauma Coalition.

**Table 3.** Key Findings of the Review.

• Trauma-informed intervention in ECEC settings is nascent but growing, with the predominant focus placed on training and coaching. The inclusion of coaching in addition to training itself was suggested to increase fidelity and strengthen outcomes. • Increasingly, programs are adopting multi-tiered system of support to address early childhood trauma, with these models suggesting promising results. • Studies focused on assessment of teacher-level outcomes. Child, organization, and caregiver-level outcomes are not explored to the same extent, with evaluation of organizational outcomes relying predominately on qualitative methods. • While trauma-informed programs have led to positive outcomes for children, teachers, classrooms, and organizations, the sustainability of program impact is unknown, and the causal mechanistic pathways are yet to be explored.

Six interventions (Head Start Trauma Smart [HSTS] [14]; Trauma Supplement Intervention [4]; Partnerships Program [13]; TIC BSC Initiative [1]; TIES [5, 7]; the New Haven Trauma Coalition [NHTC] [12]) were classified as multi-tiered because they involved more than universal workforce capacity-building, and incorporated at least one component focused on children in need of targeted support. For example, HSTS (Holmes et al., 2015) included training for all adults surrounding the child (i.e., teachers, parents, administrators, bus drivers), provided by a master’s level trained, licensed therapist. Children who may have witnessed traumatic events or experienced trauma were identified through behavior and trauma screening and referred to intensive trauma-specific supports (e.g., play therapy, sand tray). In addition, HSTS therapists provided classroom consultation for teachers and children on a need-basis, and teachers and supervisors were connected for peer-based mentoring.

Except for the 2Gen Thrive program (Woods-Jaeger et al., 2018) that provided targeted classroom-based support for children and capacity-building sessions for parents at risk of toxic stress, all interventions included in-service training that aimed to promote teachers’ awareness, knowledge, and skills relating to trauma-informed practice. In nine interventions [1–3, 5–7, 10–14], this professional development was coupled with coaching. For example, in Sunshine Circles (Tucker et al., 2017; 2021), teachers participated in training and were then coached to deliver the intervention.

Five trauma-informed programs included an organizational component, with four classified as multi-tiered (Trauma supplement intervention [4]; TIC BSC Initiative [1]; TIES [5, 7]; HNTC [12]). These typically involved training at an organization-level to promote a trauma-informed culture and coordinated implementation of trauma-informed practices. For instance, in the TIC BSC initiative [1], cross-role teams (i.e., teachers, administrators, leaders) were engaged to achieve a trauma-informed culture through distributed leadership from all levels of organizational system (Douglass et al., 2021).

An explicit program was embedded in five interventions (Sunshine Circles [10]; STRIVE [11]; 2Gen Thrive [9]; Partnerships Program [13]; TIES [5, 7]). In 2Gen Thrive, Classroom Theraplay was delivered to children by mental health therapists in partnership with teachers, whereas in Sunshine Circles, teachers received training and then delivered a program with the support of a coach. In the STRIVE program [11], the STRIVE Toolkit supplemented training, consultation, and coaching through practical tools including emotion cards, stress balls, noise-cancelling headphones, and calming scents. These tools supported children to identify and regulate their emotions. Family involvement was described in five studies, through training (2Gen Thrive [9]; HSTS [14]; TIES [5, 7]), regular meetings (TIC BSC [1]), and care coordination (NHTC [12]). Cultural sensitivity and adaptations to meet community’s needs, values, and norms were considered in four programs (2Gen Thrive [9]; HSTS [14]; Partnerships program [13]; TIC BSC Initiative [1]). For instance, in 2Gen Thrive, a diverse Community Action Board (i.e., representatives from educational organizations, social services, healthcare, and parents with ACE) was formed to identify cultural adaptations and augmentations (Woods-Jaeger et al., 2018).

### Outcomes and Measures

Seven studies examined the impact of ECEC center-based trauma-informed interventions on child outcomes, including social-emotional competence [3, 9, 10], and behavioral functioning [3, 4, 13, 14]. Teacher-level outcomes were most commonly reported, with six studies investigating teachers’ knowledge, attitudes, beliefs, and confidence [1, 2, 4, 8, 11, 12], five examining the impact on teacher behavior [1, 6, 8, 10, 12], and three studies assessing change in teacher efficacy [4, 11, 13] following the intervention. In terms of classroom-level outcomes, nine studies examined the quality of teacher–child interaction and environmental quality [4, 5, 7–11, 13, 14]. Trauma-awareness, and readiness to change, reported as organization-level outcomes, were gauged in two studies [1, 4]. Only one study assessed a parent-level outcome [9]. Details of measures and program outcomes are provided in the Supplemental Table S3.

#### Child-level outcomes

Four studies found trauma-informed intervention in ECEC significantly and positively impacted children’s behavioral outcomes [3, 4, 13, 14]. HSTS aimed to support social and cognitive development by creating an integrated, trauma-informed culture within Head Start centers. In Holmes et al. (2015), the Achenbach System of Empirically Based Assessment (Achenbach & Rescorla, 2000), a valid and reliable diagnostic tool, was used by teachers to assess children’s behavior change, while the Child Behavior Checklist was completed by parents to evaluate children’s behavioral outcomes after exposure to HSTS intervention. Both assessments were completed at time of referral, and either post-intervention or every 6 months, whichever arrived first. Teachers reported significant improvement in children’s attention, externalizing behavior, and oppositional defiance. Similarly, parents reported significant improvement in externalizing problems, attention/hyperactivity, and internalizing behaviors in home settings. The Partnerships Program, which provided trauma-informed workforce professional development, ongoing consultation, and trauma-specific interventions for identified children demonstrated similar benefits on children’s behavioral development. Data collected at baseline and post-intervention suggested significant improvement in children’s resilience (Shamblin et al., 2016).

Children’s social-emotional competence was evaluated in three studies [3, 9, 10]. Tucker et al. (2017, 2021) examined the effect of Sunshine Circles on child development using two standard measurement tools. Data collected at three time-points during the year demonstrated significant improvements in children’s social-emotional skills, behavioral regulation, problem-solving, and fine-motor control, in particular managing feelings, cooperation, accepting limits, peer-interaction, and friendship, and social problem-solving (Tucker et al., 2017, 2021). Unlike Tucker et al.’s (2017, 2021) adoption of standardized measures, Woods-Jaeger et al. (2019) conducted qualitative interviews with teachers to gauge children’s social-emotional development post-intervention (2Gen Thrive). Benefits of Classroom Theraplay include greater expressiveness, increased interaction with peers and caregivers, and initiation of activities, where children showed enjoyment and ability to initiate connections with peers and caregivers (Woods-Jaeger et al., 2019).

#### Teacher-level outcomes

With the exception of 2Gen Thrive, all interventions included teacher training and teacher-level outcomes were the most commonly assessed outcomes. Teacher knowledge, attitudes, beliefs, and confidence were assessed in seven studies, with three qualitative studies adopting either an interview [1] or focus group [8], four quantitative studies utilizing a survey method [2, 6, 11, 12], and one using direct observation of teacher practices [4]. Among the three studies that used quantitative survey methods, three relied on scales that were developed for the study [2, 6, 11]. The Attitudes Related to Trauma-informed Care measure [2] was the only psychometrically validated measure used. Teachers demonstrated increased understanding [1], awareness [1, 2, 11], knowledge [2, 4, 6, 11, 12], empathy [8], mindfulness [8], and skills [12] relating to trauma-informed practice after participating in the intervention.

Five studies examined the impact of trauma-informed interventions on teacher behavior. Qualitative data collection methods included interviews with teachers and coaches [1], and focus group discussions [8], while quantitative studies relied on teacher self-report surveys [6, 12], coach report [6], and observations [6, 10]. Roots of Resilience [6], which involved online training and video-based coaching, was associated with significant improvement in teachers’ language modeling and regard for child perspectives. Following the Sunshine Circles intervention [3, 10], where teachers received training and coaching in an attachment-based program, teachers demonstrated increased ability to communicate with children, manage their classroom, and maintain a calm and active environment to support learning (Tucker et al., 2017). Similarly, qualitative findings showed teachers’ increased use of trauma-informed strategies [1, 8], family-centered communication skills [1], self-care strategies [8], communication strategies with children [10], and classroom-management strategies [10] post-intervention.

Self-efficacy was evaluated in three studies [4, 11, 13]. The Secondary Trauma Self-Efficacy Scale (Cieslak et al., 2013), a psychometrically evaluated, standardized measure, was used by Gilles and Carlson (2020) to evaluate a Trauma Supplement Intervention using a pre-post controlled design. Intervention and control groups did not differ with regards to teachers’ self-efficacy following the intervention. However, in Shamblin et al. (2016), teachers reported significant improvement in confidence and hopefulness to impact challenging child behavior after taking part in The Partnerships Program, based on Teacher Opinion Scale (Geller & Lynch, 1999), a non-standardized, 12-item self-report measure.

#### Classroom-level outcomes

Two classroom-level outcomes were assessed in the included studies: teacher–child interaction quality and environment quality. The Classroom Assessment Scoring System (CLASS; Pianta et al., 2008) was used in six studies [4, 5, 7, 9, 11, 14]. This is a standardized, reliable, and valid observational instrument that assesses the quality of teacher–child relationships across three domains: Emotional Support, Classroom Organization, and Instructional Support (Pianta et al., 2008). Among the six studies employing this tool, two indicated a positive overall trend without providing specific statistics, following the 2Gen Thrive [9] and HSTS [14] programs. Of the remaining four studies, mixed results were reported. A significant increase in Emotional Support and Classroom Organization were identified in three studies [5, 7, 11]; however, inconsistent findings regarding Instructional Support were found, with two studies suggesting no significant differences between control and intervention groups (TIES) [5, 7] at pre- and post-intervention (STRIVE) [11], and one reporting a significant increase in this domain [5]. Interestingly, two studies that evaluated the same intervention (TIES) [5, 7] yielded different results. Rishel et al. (2019) [7] captured data from 52 classrooms and found no significant difference between intervention and control rooms in levels of Instructional Support. Tabone et al. (2020) [5] extended this evaluation using a stronger study design including matched classroom pairs and a larger sample (74 classrooms). They found significant improvement in rooms that participated in TIES compared to controls across all three CLASS domains (Tabone et al., 2020).

Only one study included a follow-up assessment. Whitaker et al. (2019) examined the ETA intervention using an online teacher survey including three outcome domains: relational capacities, health and wellbeing, relationship quality [8]. The survey was completed by teachers at baseline, post-intervention, and 5-month follow-up. Results suggested that while the impact of ETA on conflict differed significantly by teacher education level at post-intervention, this difference was no longer significant at 5-month follow-up. No other significant changes in outcome measures were reported (Whitaker et al., 2016).

#### Organization-level outcomes

Organization-level outcomes were evaluated in two studies using qualitative methods [1, 4]. An amended version of the System of Care Trauma-informed Agency Assessment (TIAA; THRIVE Evaluation Committee, 2011) was utilized for use in a Head Start preschool setting by Gilles and Carlson (2020). Six organizational-level elements were assessed: (a) physical and emotional safety, (b) trauma competence, (c) cultural competence, (d) commitment to trauma-informed philosophy, (e) trustworthiness, and (f) youth and family empowerment. Guided by the TIAA, Gilles and Carlson (2016) conducted structured interviews with informants including the director of preschool programs, Head Start Mental Health Consultants, site supervisors and teachers, both pre- and post-intervention, to explore organizational change. Findings suggested that administrators were more receptive to assessing and documenting children’s trauma history in a uniform fashion, more aware of the impact of secondary trauma, and the necessity of providing support for staff (Gilles & Carlson, 2020). Increased trauma-awareness and readiness to change were identified within the ECEC center, with staff recognizing the benefits of trauma-informed practices and implementing TIC strategies at the organizational-level (Gilles & Carlson, 2020). Similarly, Douglass et al. (2021) examined the organizational practices and system change through in-depth interviews, meeting observations, and reviewing documents such as improvement tracking forms and intranet posts, finding improvement in workplace relationships, shared leadership, collaborative learning, and interagency collaboration (Douglass et al., 2021).

#### Parent-level outcomes

Though parent-involvement was reported in five interventions, parent-level outcomes were only reported in one study. Training for parents to address intergenerational trauma was a significant component in the 2Gen Thrive [9] (Woods-Jaeger et al., 2018). A significant reduction in caregivers’ depressive symptoms and distress, measured via standardized scales was identified between pre- to post-intervention.

## Discussion

This review systematically examined the type of trauma-informed programs that have been implemented and evaluated in ECEC settings, and explored the child, teacher, caregiver, classroom, and organization-level outcomes reported. The findings suggest that trauma-informed interventions in ECEC are nascent but growing, with the focus predominantly placed on teacher training and coaching. Training was delivered through in-person and online modules that aimed to promote teachers’ awareness of trauma and its impact, support recognition of trauma-related behaviors, and provide strategies to support trauma-impacted young children. Eight interventions included both training and coaching. Coaching was delivered in-person in the ECEC setting, except for the Roots of Resilience program that relied on an online, relationship-based coaching model (Lipscomb et al., 2019). Results indicated both formats positively impacted teachers’ knowledge and practice. Coaching strategies included modeling, guided practice with feedback, and reflection. The inclusion of coaching and/or consultation is more effective than training in isolation, as specialized coaching and consultation are responsive to teachers’ needs and practices in context (Taylor et al., 2022). Coaching provides teachers with the opportunity to reflect on their practice and transfer knowledge and skills acquired through training into their practices. Combined coaching and training are likely to address challenges expressed by early childhood teachers relating to work with trauma-impacted children. For example, Lombardi (2020) claimed that teachers were overwhelmed by the quantity of strategies provided during trauma-related in-service training and faced difficulty incorporating learning into their practice. Hence, future interventions should also consider combining training and follow-up contextualized coaching to increase fidelity of the intervention and maximize effects.

Multi-tiered system of supports have not been adopted within ECEC to the same extent as other educational settings due to a variety of barriers (e.g., diverse types of early learning services with different funding sources; no universal curriculum) (Shepley & Grisham-Brown, 2019). The six trauma-informed interventions that were considered multi-tiered demonstrated promising impacts on child, teacher, classroom, and organization-level outcomes. In addition to universal Tier 1 level supports for all children (e.g., workforce training to create a supportive environment), targeted Tier 2 and/or individualized Tier 3 supports are also available in multi-tiered interventions. These holistic approaches have the potential to promote supportive early learning environments, assist teachers to identify child behaviors that may be related to traumatic experiences, and intervene early.

Among these multi-tiered programs, four included components that targeted organizational support and system change. However, only two examined organization-level outcomes, with evaluation relying on qualitative methods such as interview (Douglass et al., 2021; Gilles & Carlson, 2020). We suggest greater attention should be paid to trauma-informed organizational change in ECEC, where the infusion of trauma-informed awareness, culture, policies, and structures can support the teachers’ trauma-informed practices and enable quality improvement (Douglass, 2016). Organizational change research also suggests that individuals face barriers adopting and sustaining new practices such as trauma-informed strategies without simultaneous actions at the broader system level (Douglass, 2016; Gittell, 2016; Kania et al., 2018). Psychometrically sound measures could support early learning organizations to better understand the extent to which they are trauma-informed, and monitor effectiveness of programming. Nevertheless, our findings are aligned with Champine et al.’s (2019) systematic review, that reported system-level measurement tools, especially related to the organizational environment and practices in educational contexts, were underdeveloped.

Overall, trauma-informed interventions in ECEC have led to positive improvements in teachers’ trauma-informed knowledge, awareness, and skills (Douglass et al., 2021; Gilles & Carlson, 2020; Lipscomb et al., 2019; McConnico et al., 2016; Perry & Daniels, 2016; Shamblin et al., 2016; Whitaker et al., 2019), self-efficacy and confidence (Gilles & Carlson, 2020; McConnico et al., 2016; Shamblin et al., 2016), children’s social, emotional, and behavioral competence (Gilles & Carlson, 2020; Holmes et al., 2015; Shamblin et al., 2016; Tucker et al., 2017, 2021; Woods-Jaeger et al., 2018), the quality of teacher–child classroom interaction (Rishel et al., 2019; Tabone et al., 2020; Tucker et al., 2017; Whitaker et al., 2019; Woods-Jaeger et al., 2018), learning environments (Holmes et al., 2015; McConnico et al., 2016; Shamblin et al., 2016), organization and system change in terms of positive workplace relationships, shared leadership and collaboration (Douglass et al., 2021), and depression and distress in caregivers (Woods-Jaeger et al., 2018). However, little is known about the sustainability of outcomes over time, with only one study including a follow-up analysis that suggested no sustained impact (Whitaker et al., 2019). Meanwhile, a theory of change was reported in only one study (Lipscomb et al., 2019). This aligns with the findings of Schindler et al. (2019), who highlighted a lack of attention toward theories of change in early childhood intervention research. Similarly, studies focused on outcomes, while the causal mechanistic pathways, and how, why, for whom, and under what conditions the intervention does or does not work was generally not explored.

It is promising that cultural adaptations to meet the needs, values, and norms of the local community was considered in four programs. Such cultural adaptation is important and correlates with improved outcomes for ethnic and racial minorities (McMullen et al., 2013). In Kumpfer et al.’s (2002) evaluation of a culturally adapted version of the Strengthening Families Intervention, a 40% increase in program retention was reported. Finally, family involvement was described in five interventions. This included the provision of training materials to parents, with one program inviting parents to provide feedback on areas for improvement (Douglass et al., 2021). Though only one study evaluated parent-level outcomes, it reported a significant reduction in parental depression and distress (Woods-Jaeger et al., 2018).

## Limitations

### Limitations of the Current Review

While this is the first review to synthesize the current evidence of trauma-informed interventions systematically and comprehensively in ECEC settings, several limitations should be considered when interpreting the findings. First, inclusion criteria were limited to studies published in English after 2011, thus relevant studies published in languages other than English and prior to 2011 may have been missed. The scoping nature of the review means that the quality of evidence captured was not evaluated. Further, social and emotional learning programs may include elements such as resilience that can buffer the negative impact of trauma (Sexton et al., 2015); however, these studies were not included as this review focused on programs where trauma was the explicit core focus. Given that all studies were conducted in the United States, caution should be applied when generalizing the findings to other cultural contexts, where ECEC service provision, teacher training, and inter-disciplinary support models may vary.

### Limitations in the Evidence

Though examining the methodological quality of studies was not a focus of this scoping review, several methodological limitations in the studies emerged. First, studies tended to rely on self-report measures, leading to potential for response bias (Gilles & Carlson, 2020; Lipscomb et al., 2019; Tucker et al., 2017). The continued use of standardized measures will support a more robust and rigorous evidence-base. Second, evaluation of organization-level outcomes relied heavily on qualitative methods such as interviews (Douglass et al., 2021; Gilles & Carlson, 2020). Aligned with the finding of Champine et al.’s (2019) review and notwithstanding the deep insights that can be gleaned through qualitative research, we recognize the current gap in psychometrically sound measurement tools to assess trauma-informed system-level outcomes. Further, this review uncovered literature examining the effectiveness of ECEC trauma-informed interventions on short-term child, teacher, classroom, organization, and caregiver outcomes. However, little is known about the sustainability of such outcomes over time. Longitudinal methods are needed to better understand what works and what sustains with respect to trauma-informed intervention. This may also inform our understanding of how trauma-informed interventions affect children’s learning and developmental trajectory over the longer term, and the return on investment for prevention and early intervention for children impacted by trauma within the ECEC sector. Finally, studies generally measured the effectiveness of interventions without exploring underlying causal mechanisms. How, why, for whom, and under what conditions interventions do or do not work is yet to be explored. Methodologies such as realist evaluation could help to uncover such knowledge (Pawson & Tilley, 1997).

## Future Directions

Despite limitations of current published evidence, findings of this review uncovered knowledge that could inform the development and improvement of ECEC trauma-informed interventions (See Table 4). The integration of coaching in addition to training appears to be effective in supporting teachers to translate what they have learned into their practice. There is also value in continuing to develop and evaluate multi-tiered trauma-informed interventions, and examine the sustainability of outcomes over time. Understanding of the context in which ECEC centers operate and the cultural and socioeconomic background of the children and families they serve can help tailor trauma-informed approaches (Cole et al., 2013). Finally, partnerships between families and their ECEC centers are critical to ensuring high-quality education and care (Cottle & Alexander, 2014). Partnering with families and caregivers, and listening to their perspectives should be further considered (Arthur et al., 2021). Such partnerships have the potential to inform and improve program implementation and enhance a trauma-informed culture within the community, and subsequently maximize child outcomes.

**Table 4.** Implications for Research, Practice, and Policy.

Research Implications
• Studies tended to reply on self-report measures, leading to potential for response bias. The continued use of standardized measures will support a more robust and rigorous evidence-base. • The need to further explore the psychometrically sound measurement tools to assess trauma-informed organizational-level outcomes. • Longitudinal methods are needed to better understand what works and what sustains with respect to trauma-informed intervention. • Studies generally measured the effectiveness of interventions without exploring underlying causal mechanisms. How, why, for whom, and under what conditions interventions do or do not work is yet to be explored. Methodologies such as realist evaluation could help to uncover such knowledge.
Practice and Policy Implications
• Training should not be a “one-off” event, rather, ongoing support (e.g., coaching, consultation, supervision) is needed in supporting teachers translate what they have learned into practice. • Organizational change in ECEC should be explored, where the infusion of trauma-informed awareness, culture, policies, procedures, and structures can support the teachers implement and sustain trauma-informed practices. • Understanding of the context in which ECEC centers operate and the cultural and socioeconomic background of the children and families they serve can help tailor trauma-informed approaches. Contextual considerations and cultural adaptations are needed. • Partnerships between families and their ECEC centers have the potential to inform and improve program implementation and enhance a trauma-informed culture within the community. • Trauma-informed interventions in early childhood is still nascent. Governments should collaborate across disciplines and sectors to continue develop and scale-up evidence-based programs that support and build ECEC services’ capacity to be trauma-informed.

*Note*. ECEC = Early Childhood Education and Care.

## Conclusion

Trauma-informed intervention in ECEC settings is nascent but growing, with the predominant focus placed on training and coaching. The inclusion of coaching in addition to training itself was suggested to increase fidelity and strengthen outcomes. Increasingly, programs are adopting multi-tiered system of support to address early childhood trauma, with these models suggesting promising results. The majority of multi-tiered trauma-informed interventions embed a component that targets organization and system change. However, few evaluated organization-level outcomes, with researchers relying on qualitative methods to understand organizational impact. Further exploration of measurement tools and adoption of evaluation methods such as RCTs can enhance the evidence and potentially promote the application of trauma-informed approaches in early childhood educational settings. While trauma-informed programs have led to positive outcomes for children, teachers, classrooms, and organizations, the sustainability of program impact is unknown, and the causal mechanistic pathways have not been explored. Longitudinal studies that explore program outcomes over time, and the causal mechanism that underline impact will advance our current understanding of the effectiveness of trauma-informed interventions in early childhood education and care settings.

## Supplemental Material

sj-docx-1-tva-10.1177_15248380231162967 – Supplemental material for Trauma-informed Interventions in Early Childhood Education and Care Settings: A Scoping Review Click here for additional data file. Supplemental material, sj-docx-1-tva-10.1177_15248380231162967 for Trauma-informed Interventions in Early Childhood Education and Care Settings: A Scoping Review by Yihan Sun, Claire Blewitt, Victoria Minson, Rachael Bajayo, Lee Cameron and Helen Skouteris in Trauma, Violence, & Abuse

## References

[bibr1-15248380231162967] Achenbach T. Rescorla L. (2000). An integrated system of multi-informant assessment (pp. 74–100). University of Vermont, Research Centre for Children, Youth, & Families.

[bibr2-15248380231162967] American Psychiatric Association. (2013). Diagnostic and statistical manual of mental disorders (5th ed.). American Psychiatric Publishing.

[bibr3-15248380231162967] American Psychological Association. (2022). Trauma. https://www.apa.org/topics/trauma/index

[bibr4-15248380231162967] Arthur L. Beecher B. Death E. Dockett S. Farmer S. (2021). Programming and planning in early childhood settings. Cengage.

[bibr5-15248380231162967] Avery J. C. Morris H. Galvin E. Misso M. Savaglio M. Skouteris H. (2021) Systematic review of school-wide trauma-informed approaches. Journal of Child and Adolescent Trauma, 14, 381–397. 34471456 10.1007/s40653-020-00321-1 PMC8357891

[bibr6-15248380231162967] Briggs-Gowan M. J. Carter A. S. Clark R. Augustyn M. McCarthy K. J. Ford J. D. (2010). Exposure to potentially traumatic events in early childhood: differential links to emergent psychopathology. Journal of Child Psychology and Psychiatry, and Allied Disciplines, 51(10), 1132–1140. 10.1111/j.1469-7610.2010.02256.x 20840502 PMC3106304

[bibr7-15248380231162967] Bronfenbrenner U. Ceci S. J. (1994). Nature-nuture reconceptualized in developmental perspective: A bioecological model. Psychological Review, 101, 568–586. 7984707 10.1037/0033-295x.101.4.568

[bibr8-15248380231162967] Blaustein M. E. Kinniburgh K. M. (2018). Treating traumatic stress in children and adolescents: How to foster resilience through attachment, self-regulation, and competency. Guilford Publications.

[bibr9-15248380231162967] Cappa C. Jijon I. (2021). COVID-19 and violence against children: A review of early studies. Child Abuse & Neglect, 116(Pt 2), 105053. 10.1016/j.chiabu.2021.105053 PMC9754317 33965215

[bibr10-15248380231162967] Champine R. Lang J. Nelson A. Hanson R. Tebes J. (2019). Systems measures of a trauma-informed approach: A systematic review. American Journal of Community Psychology, 64(3–4), 418–437. 31469452 10.1002/ajcp.12388 PMC7003149

[bibr11-15248380231162967] Cieslak R. Shoji K. Luszczynska A. Taylor S. Rogala A. Benight C. C. (2013). Secondary trauma self-efficacy: Concept and its measurement. Psychological Assessment, 25, 917–928. 23647049 10.1037/a0032687

[bibr12-15248380231162967] Cole R. Hayes B. Jones D. Shah S. (2013). Coping strategies used by school staff after a crisis: A research note. Journal of Loss & Trauma, 18(5), 472. 25170309 10.1080/15325024.2012.719335 PMC4118944

[bibr13-15248380231162967] Copeland W. E. Keeler G. Angold A. Costello E. J. (2007). Traumatic events and posttraumatic stress in childhood. Archives of General Psychiatry, 64(5), 577–584. 17485609 10.1001/archpsyc.64.5.577

[bibr14-15248380231162967] Cottle M. Alexander E. (2014). Parent partnership and “quality” early years services: Practitioners’ perspectives. European Early Childhood Education Research Journal, 22(5), 637–659.

[bibr15-15248380231162967] De Bellis M. Zisk A. (2014), The biological effects of childhood trauma. Child and Adolescent Psychiatric Clinics of North America, 23(2), 185–222. 24656576 10.1016/j.chc.2014.01.002 PMC3968319

[bibr16-15248380231162967] De Prince A. P. Weinzierl K. M. Combs M. D . (2009). Executive function performance and trauma exposure in a community sample of children. Child Abuse & Neglect, 33(6),353–361. 19477515 10.1016/j.chiabu.2008.08.002

[bibr17-15248380231162967] Douglass A. (2016). Trauma and young children. In Couchenour D. Chrisman J. (Eds.), The Sage encyclopedia of contemporary early childhood education (Vol. 3, pp. 1395–1396). SAGE Publications Ltd.

[bibr18-15248380231162967] *Douglass A. Chickerella R. Maroney M. (2021). Becoming trauma-informed: A case study of early educator professional development and organizational change. Journal of Early Childhood Teacher Education, 42(2), 182–202.

[bibr19-15248380231162967] Dye H. (2018). The impact and long-term effects of childhood trauma, Journal of Human Behaviour in the Social Environment, 28(3), 381–392.

[bibr20-15248380231162967] Felitti V. J. Anda R. F. Nordenberg D. Williamson D. F. Spitz A. M. Edwards V. Marks J. S. (1998). Relationship of childhood abuse and household dysfunction of many of the leading causes of death in adults. The adverse childhood experiences (ACE) study. American Journal of Preventive Medicine, 14(4), 245–258. 9635069 10.1016/s0749-3797(98)00017-8

[bibr21-15248380231162967] Geller S. Lynch K. (1999). Teacher opinion survey.Virginia Commonwealth University Intellectual Property Foundation and Wingspan, LLC.

[bibr22-15248380231162967] *Gilles M. Carlson J. (2020). A pilot study on the effects of a supplemental trauma intervention within a head start preschool program. Research and Practice in the Schools, 7(1), 49–69.

[bibr23-15248380231162967] Gittell J. H. (2016). Transforming relationships for high performance: The power of relational coordination. Stanford University Press.

[bibr24-15248380231162967] Hanson R. F. Lang J. M. Fraser J. G. Agosti J. R. Ake G. S. Donisch K. M. Gewirtz A. H. (2018). Trauma-informed care: Definitions and statewide initiatives. In Klika J. B. Conte J. R. (Eds.), The APSAC handbook on child maltreatment (4th edn, pp. 272–291). SAGE.

[bibr25-15248380231162967] *Holmes C. Levy M. Smith A. Pinne S. Neese P. (2015). A model for creating a supportive trauma-informed culture for children in preschool settings. Journal of Child and Family Study, 24(6), 1650–1659. 10.1007/s10826-014-9968-6 PMC4419190 25972726

[bibr26-15248380231162967] Iachini A. Petiwala A. DeHart D. (2016). Examining adverse childhood experiences among students repeating the ninth grade: Implications for school dropout prevention. Children & Schools, 38, 218–227.

[bibr27-15248380231162967] Jimenez M. E. Wade R. Lin Y. Morrow L. M. Reichman N. E. (2016). Adverse experiences in early childhood and kindergarten outcomes. Pediatrics, 137(2), 1–11. 10.1542/peds.2015-1839 PMC4732356 26768347

[bibr28-15248380231162967] Kania J. Kramer M. Senge P. (2018). The waters systems change. FSG. Reimagining Social Change.

[bibr29-15248380231162967] Kempke S. Luyten P. Claes S. Wambeke P. Bekaert P. Goossens L. Houdenhove B. (2013). The prevalence and impact of early childhood trauma in chronic fatigue syndrome. Journal of Psychiatric Research, 47(5), 664–669. 23421962 10.1016/j.jpsychires.2013.01.021

[bibr30-15248380231162967] Kumpfer K. L. Alvarado R. Smith P. Bellamy N. (2002). Cultural sensitivity and adaptation in family-based prevention interventions. Prevention Science, 3, 241–246. 12387558 10.1023/a:1019902902119

[bibr31-15248380231162967] *Lipscomb S. Hatfield B. Lewis H. Goka-Dubose E. Fisher P. (2019). Strengthening children’s roots of resilience: Trauma-responsive early learning. Children and Youth Services Review, 107, 104510.

[bibr32-15248380231162967] Lombardi C. (2020). Early childhood teacher perspectives regarding preparedness to teach children experiencing trauma. [Unpublished doctoral dissertation]. Walden University.

[bibr33-15248380231162967] *McConnico N. Boynton-Jarrett R. Bailey C. Nandi M. (2016). A framework for trauma-sensitive schools: Infusing trauma-informed practices into early childhood education systems. ZERO to THREE, 36(5), 36–44.

[bibr34-15248380231162967] McMullen J. O’Callaghan P. Shannon C. Black A. Eakin J. (2013). Group trauma-focused cognitive-behavioural therapy with former child soldiers and other war-affected boys in the DR Congo: A randomised controlled trial. Journal of Child Psychology and Psychiatry, 54, 1231–1241. 23738530 10.1111/jcpp.12094

[bibr35-15248380231162967] Munn Z. Peters M. D. J. Stern C. Tufanaru C. McArthur A. Aromataris E. (2018). Systematic review or scoping review? Guidance for authors when choosing between a systematic or scoping review approach. BMC Medical Research Methodology, 18, 143. 30453902 10.1186/s12874-018-0611-x PMC6245623

[bibr36-15248380231162967] Organization for Economic Cooperation and Development [OECD]. (2021). Education at a glance 2021: OECD indicators. OECD Publishing.

[bibr37-15248380231162967] *Orapallo A. Grant B. Baker C. (2021). Examining the effectiveness of trauma smart training: Staff satisfaction, knowledge, and attitudes. Psychological Trauma: Theory, Research, Practice, and Policy, 13(8), 891–898. 34180686 10.1037/tra0001075

[bibr38-15248380231162967] *Perry D. Daniels M. (2016). Implementing trauma – informed practices in the school setting: A pilot study. School Mental Health, 8, 177–188.

[bibr39-15248380231162967] Pianta R. C. La Paro K. M. Hamre B. K. (2008). Classroom assessment scoring system (CLASS) manual, K – 3. Brookes.

[bibr40-15248380231162967] Powers A Etkin A Gyurak A Bradley B Jovanovic T. (2015). Associations between childhood abuse, posttraumatic stress disorder, and implicit emotion regulation deficits: Evidence from a low-income, inner-city population. Psychiatry, 78(3), 251–264. 26391833 10.1080/00332747.2015.1069656 PMC4705548

[bibr41-15248380231162967] *Rishel C. Tabone J. Hartneet H. Szafran K. (2019). Trauma-informed elementary schools: Evaluation of school-based early intervention for young children. National Association of Social Workers.

[bibr42-15248380231162967] Sexton M. Hamilton L. McGinnis W. Rosenblum L. Muzik M. (2015). The roles of resilience and childhood trauma history: Main and moderating effects on postpartum maternal mental health and functioning. Journal of Affective Disorders, 15(174), 562–568. 10.1016/j.jad.2014.12.036 PMC4339466 25560192

[bibr43-15248380231162967] *Shamblin S. Graham D. Bianco J. (2016). Creating trauma-informed schools for rural Appalachia: The partnerships program for enhancing resiliency, confidence and workforce development in early childhood education. School Mental Health, 8, 189–200.

[bibr44-15248380231162967] Shepley C. Grisham-Brown J. (2019). Multi-tiered system of support for preschool-aged children: A review and meta-analysis. Early Childhood Research Quarterly, 47, 296–308.

[bibr45-15248380231162967] Schindler H. McCoy D. Fisher P. Shonkoff J. (2019). A historical look at theories of change in early childhood education research. Early Childhood Research Quarterly, 48, 146–154.

[bibr46-15248380231162967] Shonkoff J. P. Garner A. S Siegel B. S. ; Committee on Psychosocial Aspects of Child and Family Health; Committee on Early Childhood, Adoption, and Dependent Care; Section on Developmental and Behavioral Pediatrics (2012). The lifelong effects of early childhood adversity and toxic stress. Pediatrics, 129, 232–46. 10.1542/peds.2011-2663 22201156

[bibr47-15248380231162967] Shonkoff J. P. Phillips D. A. (Eds.). (2000). From neurons to neighbourhoods: The science of early childhood development. National Academy Press. 25077268

[bibr48-15248380231162967] Substance Abuse and Mental Health Services Administration (2014). SAMHSA’s concept of trauma and guidance for a trauma-informed approach.

[bibr49-15248380231162967] Taylor H. McCorkle L. Vestal A. Wood C. (2022). “I need you to show me:” Coaching early childhood professionals. Early Childhood Education Journal, 50(3), 503–513.

[bibr50-15248380231162967] *Tabone J. Rishel C. Hartnett H. Szafran K. (2020). Examining the effectiveness of early intervention to create trauma-informed school environments. Children and Youth Services Review, 113, 104998.

[bibr51-15248380231162967] Thomas M. S. Crosby S. Vanderhaar J. (2019). Trauma-informed practices in schools across two decades: An interdisciplinary review of research. Review of Research in Education, 43(1), 422–452. 10.3102/0091732X18821123

[bibr52-15248380231162967] THRIVE Evaluation Committee. (2011). System of care trauma-Informed agency assessment overview.

[bibr53-15248380231162967] Tricco A. C. Lillie E. Zarin W. O’Brien K. K. Colquhoun H. Levac D. Moher D. Peters M. D. J. Horsley T. Weeks L. Hempel S. Akl E. A. Chang C. McGowan J. Stewart L. Hartling L. Aldcroft A. Wilson M. G. Garritty¸ C. . . . Straus S. E. (2018). PRISMA extension for scoping reviews (PRISMA-ScR): Checklist and explanation. Annals of Internal Medicine 169(7), 467–473. 30178033 10.7326/M18-0850

[bibr54-15248380231162967] *Tucker C. Schieffer K. Lenz S. Smith S. (2021). Sunshine circles: Randomized controlled trial of an attachment-based play group with preschool students who are at risk. Journal of Child and Adolescent Counselling, 7(3), 161–175.

[bibr55-15248380231162967] *Tucker C. Schieffer K. Wills T. J. Hull C. Murphy Q. (2017, June 8). Enhancing social-emotional skills in at-risk preschool students through theraplay based groups: The sunshine circle model. International Journal of Play Therapy. Advance online publication. 10.1037/pla0000054

[bibr56-15248380231162967] *Whitaker R. Herman A. Dearth-Wesley T. Smith H. Burnim S. Myers E. Saunders A. M. Kainz K. (2019). Effect of a trauma-awareness course on teachers’ perceptions of conflict with preschool-aged children from low-income urban households: A cluster randomized clinical trial. JAMA Network Open, 2(4), e193193. 10.1001/jamanetworkopen.2019.3193 PMC6487571 31026037

[bibr57-15248380231162967] *Woods-Jaeger B. Sexton C. Gardner B. Siedlik E. Slagel L. Tezza V. O’Malley D. (2018). Development, feasibility, and refinement of a toxic stress prevention research program. Journal of Child and Family Studies, 27, 3531–3543.

