# Supplementary material for: Trauma-informed Interventions in Early Childhood Education and Care Settings: A Scoping Review
Source: Trauma Violence Abuse. 2023 Apr 12;25(1):648–62. doi: 10.1177/15248380231162967 (PMC10666515; doi:10.1177/15248380231162967)
Supplement: sj-docx-1-tva-10.1177_15248380231162967 – Supplemental material for Trauma-informed Interventions in Early Childhood Education and Care Settings: A Scoping Review [file sj-docx-1-tva-10.1177_15248380231162967.docx]

**Table S1**

*Search Terms*

| Terms |
| --- |
| trauma‐informed OR trauma‐sensitive OR “trauma services” OR trauma* OR stress OR PTSD OR “post‐traumatic stress disorder” OR violence OR abuse OR neglect OR assault OR “family breakdown” OR grief OR “mental illness” OR “substance addiction”  AND  “early learning centre” OR “early learning center” OR preschool* OR “pre school” OR “pre-school” OR childcare OR “child?care” OR kinder* OR “pre?kindergarten” OR “pre-K” OR “pre K” OR “day care” OR daycare OR “Head Start” OR “HeadStart”  AND  evaluation OR intervention OR program OR consult* OR service |

**Table S2**

*Inclusion and Exclusion Criteria*

| **Criteria** | **Included** | **Excluded** |
| --- | --- | --- |
| Type of Study | - All study designs (quantitative, qualitative, mixed-methods) were included as long as they assessed some form of intervention outcome. | - Study did not assess a child, educator, room or organizational level outcome post-intervention. |
| Participants | - Centre-based Early Childhood Education and Care, including kindergartens, preschools, and childcare services for children from birth to 6 years of age. | - After school-hours care. - School settings (i.e., primary school, secondary school, high school) |
| Type of Intervention | - Trauma-informed approach or trauma-specific intervention, including program, framework, model or other type of intervention delivered in ECEC. - Explicit focus on trauma-informed practice. | - Program was not explicitly reported as trauma-informed (e.g., Universal SEL programs that do not draw on TIC principles or practices). |
| Outcome Measures | - At least one child, teacher, classroom or organisational level outcome assessed following the intervention | - Does not report a child, teacher, classroom or organisational-level outcome post-intervention. |
| Publication Status | - Peer-reviewed publications and dissertations, published in English between 2011 and 2021 (*note:* addition of grey literature). | - Articles in languages other than English. |

**Table S3**

*Intervention outcomes and measures*

|  | **Outcome name** | **First author (year)** | **Measures (Informant)** | **Data collection time points** | **Key findings** |
| --- | --- | --- | --- | --- | --- |
| **Teacher-level outcomes** | Teacher knowledge, attitudes, beliefs | Douglass (2021) | In-depth interviews with team members (44) and coaches (9) | Mid- and post-intervention | Educators reported expanded understanding and awareness of what it means to be trauma informed, greater empathy for children, families, and staff and increased confidence, empowerment, and teacher leadership. |
|  |  | Orapallo (2021) | Knowledge: Learning check survey with 3 multiple-choice, quiz-style questions.  Attitudes: The Attitudes Related to Trauma-Informed Care measure. | Knowledge: Post-intervention  Attitudes: Baseline, post-intervention | Knowledge: mean score across all modules was 90% correct; overall module scores ranged from 85% to 97%.  Attitudes: Statistically significant pre- to post-test increases in favourable attitudes toward trauma-informed approaches. |
|  |  | Gilles (2020) | Direct observation of teacher practices | Baseline, Post-intervention | Teachers reported positive effects of the intervention on their knowledge regarding trauma-informed care. |
|  |  | Whitaker (2019) | Focus group | 5-month post-intervention | Teachers reported greater empathy, emotional regulation, mindfulness, improved attitudes to trauma-informed care. |
|  |  | McConnico (2016) | Teacher questionnaire | Baseline, Post-intervention | Increased knowledge on the effect of trauma on children’s development (56% - 80%), awareness of the effect of trauma on children’s behaviours (75% - 90%). No significant increase in knowledge of available resources from baseline (56%) to follow-up (60%). |
|  |  | Perry (2016) | PD Satisfaction survey | Post-intervention | 16% teachers indicated being able to recognize trauma better, 47% indicated having a new technique to use with their students that will reduce stress in the classroom. |
|  | Teacher behaviour | Douglass (2021) | In-depth Interviews with team members (44) and coaches (9) | Mid- and post-intervention | Greater use of social and emotional teaching practices and family-centred communication. |
|  |  | Lipscomb (2019) | Self-report teacher survey + Coach report + CLASS Observation | Baseline, Post-intervention | - Descriptive data indicate a possible increase in regard for student perspective (Mpre = 3.90, Mpost = 4.50), and language modelling (Mpre = 2.40, Mpost = 3.30).  - Despite limited statistical power, the increase in Language Modelling was statistically significant (t (4) = 2.99, *p* = .031). |
|  |  | Whitaker (2019) | Focus group | 5-month post-intervention | Teachers reported acquired skills in responding compassionately to challenging situations; increased ability to step back, maintain a calm presence, and avoid interpreting the challenging behaviours of children or parents; ability to self-care, and create emotionally safe classrooms (less conflict, more nurturance and support). |
|  |  | Tucker (2017) | Teaching Pyramid Observation Tool for Preschool Classrooms (TPOP) (Fox et al., 2008) | Post-intervention | Structured teacher observation indicated improvement in teacher classroom performance. In terms of how well teachers communicate with students, manage the classroom, and maintain a calm, active classroom environment for learning, the differences between the control and intervention teachers were highly significant on the second and third sets of questions for the observers. The effect sizes of both were large, at *r* = .71 and .80, respectively. |
|  |  | Perry (2016) | PD Satisfaction survey | Post-intervention | 38% indicated making changes around implementing better self-care strategies. |
|  | Self-efficacy | Gilles (2020) | Secondary Trauma Self-Efficacy Scale (STSES) | Baseline, Post-intervention | No significant difference between IG and CG. However, mean STSES ratings across conditions at baseline were significantly lower four months following the end of the intervention (M=5.59 [.54]) than at post-test (M=5.79]), t (5) =3.07, p<.05. |
|  |  | McConnico (2016) | Teacher questionnaire | Baseline, Post-intervention | Teachers endorsed higher self-efficacy and confidence, in terms of preparedness (44% - 60%). |
|  |  | Shamblin (2016) | Teacher Opinion Scale | Baseline, Post-intervention | Significant pre-improvement/post-improvement in teacher confidence and hopefulness in positively impacting challenging child behaviours (*p* = 0.030). |
| **Child-level outcomes** | Social-emotional Competence | Woods-Jaeger (2018) | In-depth interview with teachers | Post-intervention | Teachers reported benefits of the Classroom Theraplay, including improvements in children’s expressiveness (from flat to expressive), increased interaction with caregivers and peers, and spontaneous singing of and initiation of activities, which demonstrated enjoyment and growing ability to initiate connections with caregivers and peers. |
|  |  | Tucker (2021), (2017) | - The Creative Curriculum GOLD (GOLD): Social emotional section (by teachers)  - Ages and Stages Questionnaire Third Edition (ASQ): Social emotional section (by parents)  - The Preschool Behaviour Questionnaire (PBQ) (by teachers)  - The Devereux Early Childhood Assessment for Preschool children (by parents) | 2017 study: 3 times a year (October, February, May)  2021 study: 3 times a year (September, January, May) | 2017 study:  - Children showed significant improvements in social-emotional skills, behavioural regulation, problem-solving, and fine motor control.  - Specific improvements occurred in domains of managing feelings, cooperation, accepting limits, peer interactions and friendships, and solving social problems.  2021 study:  - Children in intervention group showed significant increase in communication skills, problem-solving skills, personal-social skills, self-regulation skills than control group. |
|  | Behaviour | Tucker (2021) | The Devereux Early Childhood Assessment for Preschool children (DECA-P) | 3 times a year (September, January, May) | - Statistically significant differences between groups were detected for increased scores on the DECA Behaviour Concerns scale.  - Although all participants in the SC group tended to demonstrate greater intervention effects, girls tended to exhibit greater reductions in behavioural problems over time (significant). |
|  |  | Gilles (2020) | Teacher and caregiver ratings of child behaviour using the Devereux Early Childhood Assessment Preschool Program (DECA-P2, TPF and BC Scale)  The Behaviour Assessment Scale for Children, 2nd edition Progress Monitor (BASC-2 PM, Internalizing and ADHD/Externalizing Scale) |  | - No significant differences between groups in externalising, attention deficit hyperactivity disorder, and protective factors, however significant differences between groups on combination of child's protective factors,  behavioural concerns and internalising and externalising behaviours. Effects evident for all children, irrespective of trauma history.  - When caregiver ratings of symptom severity were considered, the greatest improvements were made by children who had experienced the most severe trauma. |
|  |  | Shamblin (2016) | The Devereux Early Childhood Assessment (DECA; standardized; assessed by teachers)  the Initiative, Attachment and Self-Control subscales | Baseline, Post-intervention | Compared to children in the other two ECMH programs, children in the spring Partnerships program demonstrated significantly higher resilience scores (*p* < .001). |
|  |  | Holmes (2015) | Achenbach (Teacher Report Form) and Child Behaviour Checklist (CBCL; parent) | At time of referral, Post-intervention/ Every 6 months | Significant improvement in attention, externalising behaviour, oppositional defiance based on teacher report (*p* < .05).  Parents reported significant improvements (*p* < .05) in externalising problems and attention/hyperactivity, and internalising behaviours. |
| **Classroom-level outcomes** | Interaction and environment quality | Rishel (2019) | The Classroom Assessment Scoring System (CLASS) | Baseline, Post-intervention | - IG classrooms demonstrated a significant increase from baseline to the end of school year in emotional support (*p* = 0.00), classroom organisation (*p* = 0.00), whereas no such improvements were observed in CG classrooms.  - Compared to CG classrooms, statistically significant difference was found in emotional support (*p* = 0.00) and classroom organisation (*p* = 0.00), the difference in instructional support was not significant. |
|  |  | Tabone (2020) | CLASS | Baseline, Post-intervention | - IG classrooms demonstrated a significant increase in emotional support (*p* = 0.00), classroom organisation (*p* = 0.00) from baseline to end of school year improvements in promoting trauma-sensitive classroom environments from baseline to post intervention, with no such improvements in CG.  - Compared to CG, IG classrooms showed significantly higher scores regarding emotional support (F=13.07, *p* = 0.00), classroom organisation (F = 5.52, *p* = 0.02), instructional support (F = 4.21, *p* = 0.04) after adjusting for baseline scores. |
|  |  | Whitaker (2019) | Online teacher survey:  12 constructs from validated self-report items and instruments compiled into the survey to assess relational capacities, health and wellbeing, and relationship quality. | Baseline, Post-intervention, 5-month follow-up | - No statistically significant effects of the intervention on relational trust with other adults, relational capacity, health and wellbeing.  - The effect of the intervention on conflict scores differed significantly by teacher education level. Teacher with less than a bachelor’s degree had significantly more conflict, while those with more education tended to have less conflict after taking the course (*p* = 0.01). This is no longer significant at 5-month follow-up. |
|  |  | Woods-Jaeger (2018) | CLASS | Baseline, Post-intervention | Rating scores demonstrated improvements across caregiving dimensions from baseline to post-intervention. These scores trended toward the upper end of the middle range among infants (scores of 3 to 5 reflect a “mix of effective and ineffective interaction”) and toward the highest category of effective teacher–child interactions among toddlers (scores of 6 to 7 reflect consistently observed effective teacher-child interactions). |
|  |  | Tucker (2017) | Interview with teachers | Post-intervention | Interviews with teachers confirmed intervention subjectively increased classroom cohesion, improved teacher–student relationships and improved overall classroom behaviour. |
|  |  | Gilles (2020) | CLASS | Baseline, Post-intervention | No significant difference. Teachers in both the IG and CG were rated moderately on the Emotional Support Domain of the CLASS both at baseline (intervention M=4.79 [0.26]; comparison M=5.65 [0.45]) and post-test (intervention M=5.78 [0.50]; comparison M=5.51 [0.35]). |
|  |  | McConnico (2016) | - CLASS  - Teacher questionnaire | 1) Baseline, several months post intervention  2) Baseline, Post-intervention | - Significant differences in Educational Support (p = 0.0002) and Classroom Organisation (p = 0.003). Significant differences in sub-dimension regarding Respect for Student Perspective (p = 0.0002), Positive (p = 0.0015) and Negative Classroom Climate (p = 0.0078), and productivity. No significant differences in the Instructional Support domain or sub-dimensions.    - The toolbox helped created a more compassionate and accepting classroom climate. |
|  |  | Shamblin (2016) | the Preschool Mental Health Climate Scale (observation rating system) | Baseline, Post-intervention | Significant reduction from pre-intervention scores to post-intervention scores in terms of the Negative Attributes (*p* = 0.004). |
|  |  | Holmes (2015) | CLASS (HSTS therapists – certified CLASS observer) | Baseline, later in the same year and twice in each subsequent year. | Overall trend in preferred direction over a 2-year period. |
| **Organisation-level outcomes** | Trauma-awareness, readiness to change | Douglass (2021) | - Meeting (17) and learning session (28) observations  - In-depth Interviews with team members (44) and coached (9)  - Documents (i.e., improvement tracking forms, monthly metrics, intranet posts) | Mid- and post-intervention | More positive workplace relationships and shared leadership, collaborative learning and use of data and improved interagency collaboration. |
|  |  | Gilles (2020) | Trauma-Informed Agency Assessment (TIAA, used to guide structured interviews) | Baseline, Post-intervention | - Upper-level administrators were receptive to taking into assessing and documenting children’s trauma histories in a uniform fashion.  - Administrative staff identified being more aware of the impact of secondary trauma, and the necessity of providing supports for staff.  - The agency had begun to recognize the benefit of trauma-informed practice and was taking steps towards implementing these practices across the agency. |
| **Parent-level outcomes** | Caregiver mental health (Depression & Distress) | Woods-Jaeger (2018) | - The Patient Health Questionnaire (PHQ-9)  - The Difficulties in Emotion Regulation Scale (DERS)  - Parenting Stress Index (PSI/SF) | Baseline, Post-intervention | Following the intervention, mean levels of depressive symptoms significantly decreased among group completers (p < 0.05), dropping from a mean (SD) PHQ-9 total score of 6.0 (5.0) to 4.13 (4.2) (p = 0.049). The PSI Parental Distress subscale and DERS total score decreased significantly from pre- to post-intervention (p < 0.05). |
